# Silencing of *Dihydroflavonol 4-reductase* in Chrysanthemum Ray Florets Enhances Flavonoid Biosynthesis and Antioxidant Capacity

**DOI:** 10.3390/plants11131681

**Published:** 2022-06-24

**Authors:** Sun-Hyung Lim, Da-Hye Kim, Jae-A Jung, Nam-In Hyung, YeoJin Youn, Jong-Yeol Lee

**Affiliations:** 1 Division of Horticultural Biotechnology, School of Biotechnology, Hankyong National University, Anseong 17579, Korea; kimdh143@naver.com; 2 Research Institute of International Technology and Information, Hankyong National University, Anseong 17579, Korea; 3 Floriculture Research Division, National Institute of Horticultural and Herbal Science, Rural Development Administration, Wanju 55365, Korea; jabisung@korea.kr; 4 Department of Plant and Food Sciences, Sangmyung University, Cheonan 31066, Korea; nihyung@smu.ac.kr (N.-I.H.); yjyoun01@smu.ac.kr (Y.Y.); 5 National Academy of Agricultural Science, Rural Development Administration, Jeonju 54874, Korea; jy0820@korea.kr

**Keywords:** DFR, flavonoid, metabolic flux, post-transcriptional gene silencing

## Abstract

Flavonoid biosynthesis requires the activities of several enzymes, which form weakly-bound, ordered protein complexes termed metabolons. To decipher flux regulation in the flavonoid biosynthetic pathway of chrysanthemum (*Chrysanthemum morifolium* Ramat), we suppressed the gene-encoding dihydroflavonol 4-reductase (DFR) through RNA interference (RNAi)-mediated post-transcriptional gene silencing under a floral-specific promoter. Transgenic *CmDFR*-RNAi chrysanthemum plants were obtained by *Agrobacterium*-mediated transformation. Genomic PCR analysis of *CmDFR*-RNAi chrysanthemums propagated by several rounds of stem cuttings verified stable transgene integration into the genome. *CmDFR* mRNA levels were reduced by 60–80% in *CmDFR*-RNAi lines compared to those in wild-type (WT) plants in ray florets, but not leaves. Additionally, transcript levels of flavonoid biosynthetic genes were highly upregulated in ray florets of *CmDFR*-RNAi chrysanthemum relative to those in WT plants, while transcript levels in leaves were similar to WT. Total flavonoid contents were high in ray florets of *CmDFR*-RNAi chrysanthemums, but flavonoid contents of leaves were similar to WT, consistent with transcript levels of flavonoid biosynthetic genes. Ray florets of *CmDFR*-RNAi chrysanthemums exhibited stronger antioxidant capacity than those of WT plants. We propose that post-transcriptional silencing of *CmDFR* in ray florets modifies metabolic flux, resulting in enhanced flavonoid content and antioxidant activity.

## 1. Introduction

Chrysanthemum (*Chrysanthemum morifolium* Ramat) belongs to the Asteraceae family and is an economically important floricultural crop as well as a medicinal plant species. Chrysanthemum was cultivated as a medicinal herbal plant in China in approximately the 15th century BC and subsequently spread around the world. Its use as cut flowers, potted plants, and groundcover is determined by the growth habit and cultivation of specific types. Chrysanthemum shows huge variation in flower traits including color, shape, architecture, and flowering time, owing to its genetic diversity and high ploidy [1,2]. New types with altered flower pigments will have high value for floriculture; moreover, types with improved antioxidant activities may have high value for medicinal uses.

Flavonoids are an important class of secondary metabolites widely found in plants, contributing to plant growth and development, and having prominent applications in food and medicine [3]. Flavonoids comprise flavones, flavonols, proanthocyanidins, and anthocyanins. Anthocyanins are responsible for flower colors ranging from pale pink to blue in many ornamental plants including chrysanthemum, dahlia (*Dahlia variabilis*), freesia (*Freesia hybrid*), snapdragon (*Antirrhinum majus*), and torenia (*Torenia hybrid*). The ray florets of chrysanthemum flowers can be white, yellow, green, pink, red, purple, or various bicolored forms owing to the accumulation of different proportions of anthocyanins and carotenoids [4]. There are three basic types of anthocyanins: pelargonidin, cyanidin, and delphinidin; however, the ray florets of chrysanthemum only accumulate cyanidin derivatives [4,5,6]. Unlike chrysanthemums with colored ray florets, chrysanthemums with white ray florets accumulate colorless flavones and flavonols [7]. The mechanisms of anthocyanin biosynthesis have been extensively investigated and most structural and regulatory genes involved in this process have been identified in several plant species [8,9,10,11,12,13,14].

Flavonoid biosynthesis in various plant species is associated with weakly bound multi-enzyme complexes, known as flavonoid metabolons [15,16]. Differences in spatial and temporal physical interactions between flavonoid biosynthetic enzymes result in the diverse classes of flavonoids in different tissues. Several studies have suggested that the membrane-anchored cytochrome P450s, flavone synthase (FNS), or isoflavone synthase (IFS) tether cytoplasmic flavonoid enzymes, channeling metabolites toward the biosynthesis of specialized classes of flavonoids [12,13,17]. FNS, dihydroflavonol 4-reductase (DFR), and flavonol synthase (FLS) compete for common substrates, directing the biosynthesis of flavones, anthocyanins, and flavonols, respectively. Silencing *FNS* in dahlia enhanced anthocyanin contents and overexpressing *FNS* in celery (*Apium graveolens*) decreased anthocyanins [10,18]. Additionally, experiments ectopically expressing *DFR* or *FLS* revealed an inverse correlation between flavonol and anthocyanin contents, resulting in modifications of flower color [19,20,21]. Therefore, alteration of the flavonoid biosynthetic pathway modifies metabolic flux and flavonoid contents [19,20,21].

DFR is the committed step enzyme for anthocyanin and proanthocyanidin in flavonoid biosynthesis. Several studies reported that mutations in the *DFR* gene result in the absence of pigmentation in flower and seed coats [8,19]. However, the studies on modulating flavonoid biosynthesis by *DFR* suppression have not been well characterized. To decipher the role of CmDFR in flavonoid biosynthesis, we generated transgenic chrysanthemum harboring an RNA interference (RNAi) construct that suppresses multiple chrysanthemum *CmDFR* genes and expressed this construct under the control of a flower-specific promoter. Transgenic chrysanthemum plants showed stable transgene integration throughout long-term cultivation and vegetative propagation, and no visible differences from the wild-type (WT) plants during the growth stage. Expression of *CmDFR* was successfully downregulated in ray florets of transgenic chrysanthemums, but was not affected in leaves. The total flavonoid content was higher in ray florets of transgenic chrysanthemums than in those of WT plants, resulting in greater antioxidant activity. Therefore, our results show that manipulation of CmDFR can affect flavonoid contents and antioxidant activity in chrysanthemum.

## 2. Results

### 2.1. Transgenic Chrysanthemum Harboring CmDFR-RNAi Constructs

**Figure 1:**
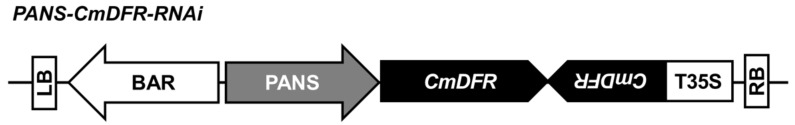
Schematic representation of the binary vector used for silencing *CmDFR* through RNA interference under the tobacco (*Nicotiana tabacum*) floral-specific promoter *PANS*. LB: left border, RB: right border, BAR: *bialaphos* resistance gene.

It is difficult to allow recombination between desirable alleles in the majority of cultivated chrysanthemums due to the hexaploid or aneuploidy genomes; therefore, genetic modifications may provide a useful tool to generate novel cultivars with desirable characters. However, obtaining a desirable phenotype by genetic modification may require the downregulation of more than one gene of interest, particularly in polyploid plants. Indeed, the resource of the National Center for Biotechnology Information (NCBI) database has seven *CmDFR* genes, which showed 96–98% identity at the nucleotide level (Supplementary Figure S1). To simultaneously down-regulate these seven *CmDFR* genes, we constructed a *PANS-CmDFR*-*RNAi* vector (Figure 1) based on *CmDFR* cDNA sequences (Supplementary Figure S1) and using the promoter of the tobacco (*Nicotiana tabacum*) flower-specific gene *PANS*.

**Figure 2:**
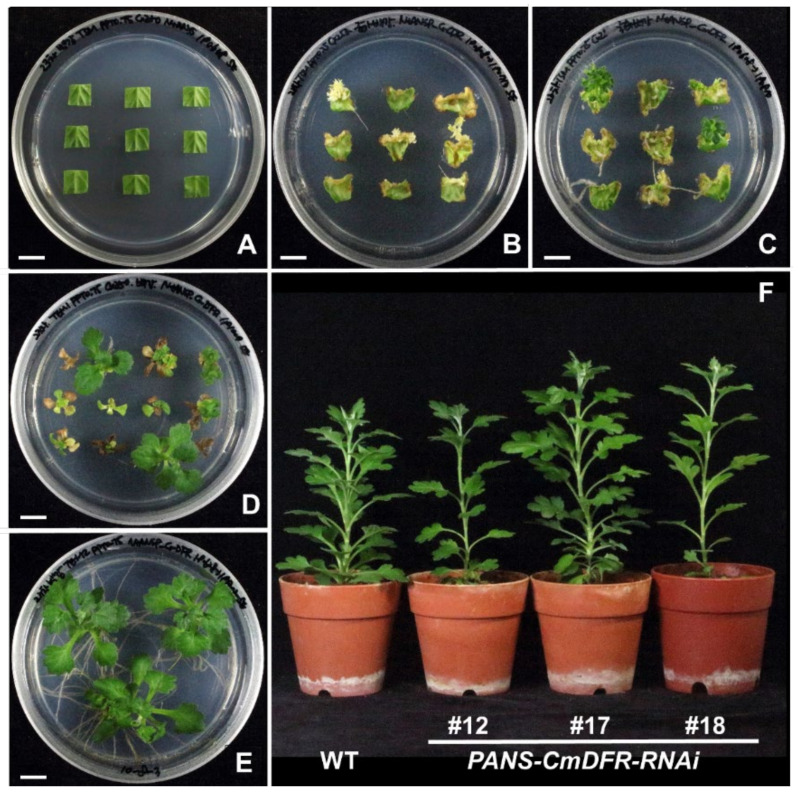
*Agrobacterium*-mediated transformation and regeneration of transgenic plants of chrysanthemum ‘Baekgang’. Leaf segments were inoculated with *Agrobacterium* harboring *PANS-CmDFR-RNAi* (**A**). After 5 weeks of culture in the dark, etiolated shoots were regenerated from explants (**B**); after 4 weeks under 16-h light/8-h dark photoperiod, etiolated shoots turned green (**C**). After 12–16 weeks of cultivation with PPT, a few shoots survived and developed (**D**). Surviving shoots were transferred into root-inducing medium with PPT (**E**). Plantlets were acclimatized and developed into normal plants (**F**). Bar = 1 cm.

Chrysanthemum transformation was performed via the leaf-disc method with *in vitro* cultured plantlets as described previously [22]. Chrysanthemum leaf discs obtained from cultured plants were submerged in *Agrobacterium* mixture (Figure 2A). Etiolated explants emerged on shoot-inducing medium containing phosphinothricin (PPT) under dark cultivation for 5 weeks and turned into green shoots under light cultivation for 4 weeks (Figure 2B,C). Putative transgenic chrysanthemum plants survived and produced roots under subsequent incubation with PPT and were transferred into pots after 2 weeks of acclimation (Figure 2D,E). The putative transgenic chrysanthemum plants were similar to wild-type (WT) plants (Figure 2F).

### 2.2. Stable Transgene Inheritance after Several Rounds of Stem Cutting Propagation

**Figure 3:**
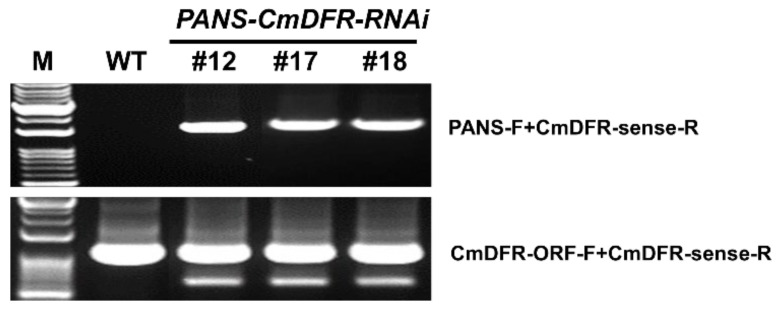
Genomic PCR analysis verified that transgenes were stably transferred into stem cuttings. DNA from wild-type (WT) and transgenic chrysanthemum plants with *PANS-CmDFR-RNAi* were subjected to amplification reactions using a primer set (PANS-F and CmDFR-sense-R) for the confirmation of the presence of the transgene. Primers amplifying the endogenous *CmDFR* gene were used as an internal control for chrysanthemum genomic DNA. M: DNA size marker of 1 Kb plus 100 bp DNA ladder.

Chimerism is common during plant transformation and affects gene transfer to plants produced by vegetative propagation from the initial transformants [23]. Therefore, precise selection of transgenic plants is indispensable for efficient and reliable transformation. Indeed, several studies have reported transgenic chrysanthemum plants that are chimeric [24,25]. To verify stable transformation in our *PANS-CmDFR-RNAi* plants, we propagated putative transgenic chrysanthemum plants via several rounds of stem cutting. We performed the PCR for detecting stable transgene integration in the genome (Figure 3). It showed that all putative transgenic chrysanthemum contained the presence of transgene fragments, about 1700 bp in length, but WT did not. In parallel, endogenous *CmDFR* sequence was amplified from all chrysanthemum plants, about 1000 bp in length.

**Figure 4:**
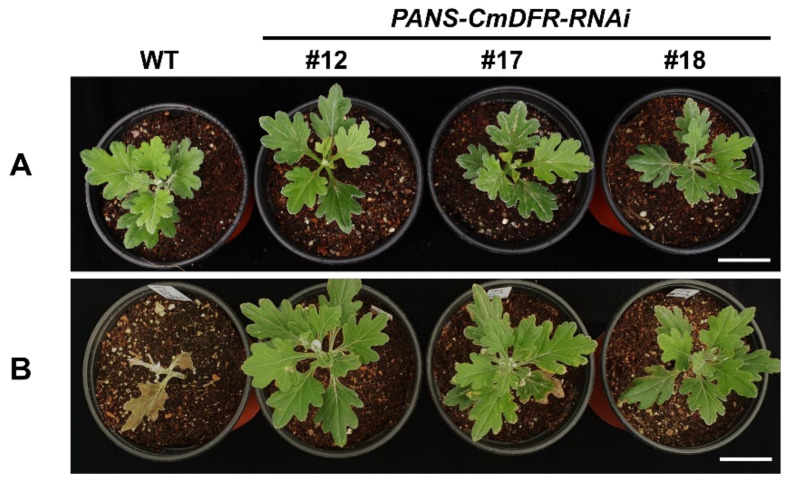
Effects of foliar spraying of 0.3% (*v*/*v*) Basta herbicide on putative transgenic chrysanthemum plants that were generated by several rounds of stem cutting propagation. Before (**A**) and after (**B**) herbicide application. The photos were taken 7 days after herbicide treatment. Bar = 4 cm.

In addition to the PCR assays, we also tested for herbicide tolerance in the propagated *PANS-CmDFR-RNAi* transgenic plants. When sprayed with 0.3% (*v*/*v*) Basta, leaves of WT plants turned yellow and rapidly dried, and the apical parts of plants fell, indicating a lack of resistance to the herbicide; however, transgenic plants appeared healthy and showed no malformation (Figure 4). Taken together, the PCR and herbicide tolerance results showed that selected transgenic chrysanthemum plants used in this method stably transfer transgene to subsequent vegetatively propagated stem-cutting plantlets without chimerism.

### 2.3. Transcript Levels of Flavonoid Biosynthetic Genes are Altered in Ray Florets of PANS-CmDFR-RNAi Chrysanthemums

The effect of RNAi-based gene silencing of *CmDFR* on the flavonoid biosynthetic pathway was evaluated by performing quantitative real-time polymerase chain reaction (qRT-PCR) analysis using ray florets and leaves. Relative to ray florets, the expression level of flavonoid biosynthetic genes, except for *chalcone isomerase* (*CmCHI*), *flavanone 3-hydroxylase* (*CmF3H*), and *anthocyanidin synthase* (*CmANS*), was dramatically lower in the leaves of both of WT and *PANS-CmDFR-RNAi* plants.

**Figure 5:**
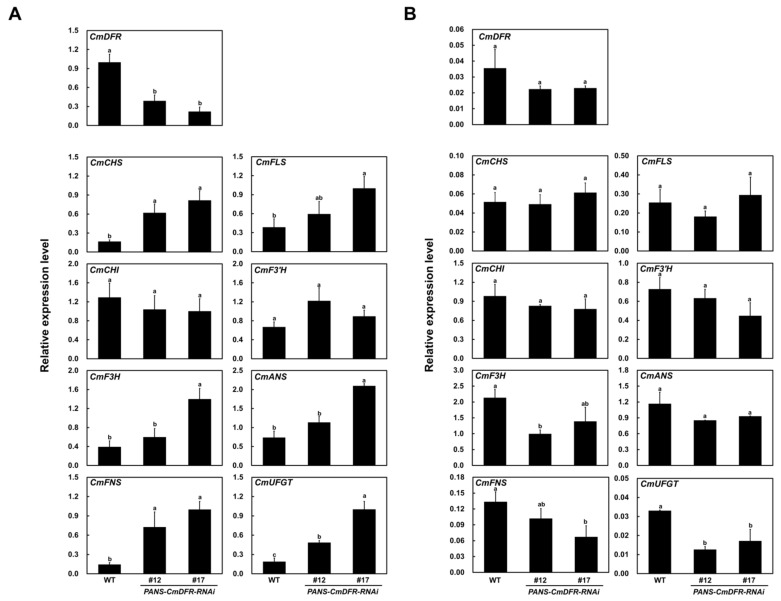
Expression of flavonoid biosynthetic pathway genes in WT and *PANS-CmDFR-RNAi* transgenic chrysanthemum ray florets (**A**) and leaves (**B**). Biosynthetic pathway genes evaluated: early biosynthetic genes *chalcone synthase* (*CmCHS*), *chalcone isomerase* (*CmCHI*), *flavanone 3-hydroxylase* (*CmF3H*), *flavone synthase* (*CmFNS*), *flavonol synthase* (*CmFLS*), and *flavonoid 3′-hydroxylase* (*CmF3′H*), and the late biosynthetic genes *dihydroflavonol 4-reductase* (*DFR*), *anthocyanidin synthase* (*CmANS*), and *UDP-glucose: flavonoid 3-O-glucosyltransferase* (*CmUFGT*). The chrysanthemum *elongation factor 1α* (*CmEF1α*) gene was used as a reference gene to normalize target gene expression. All results represent mean values ± SD from three independent biological replicates. Different letters above the bars indicate significantly different values (*p* < 0.01, one-way ANOVA followed by Duncan’s multiple range test).

As expected, qRT-PCR using primers that detect transcripts from all the annotated *CmDFR*s showed that transcript levels of *CmDFR* in ray florets of *PANS-CmDFR-RNAi* plants were 60–80% lower than those of WT plants (Figure 5A). Interestingly, the expression of all flavonoid biosynthetic genes except *CmCHI* was significantly higher in the ray florets of *PANS-DFR-RNAi* lines compared with those of WT plants. Despite *CmDFR* suppression, the early biosynthetic genes encoding chalcone synthase (CmCHS), CmFNS, CmF3H, CmF3′H, and CmFLS, as well as the late biosynthetic genes encoding CmANS and UDP-glucose: flavonoid 3-O-glucosyltransferase (CmUFGT) were expressed at dramatically higher levels in ray florets of *PANS-DFR-RNAi* than in WT ray florets. This showed that the silencing of *CmDFR* under the control of a flower-specific promoter effectively diminishes the expression of *CmDFR* in ray florets and affects the expression of flavonoid biosynthetic genes.

Although we observed strong effects on *CmDFR* expression in ray florets, the expression of *CmDFR* was not significantly different in leaves of WT and *PANS-CmDFR-RNAi* plants (Figure 5B). Moreover, flavonoid biosynthetic genes were expressed at very low levels in leaves compared with ray florets and showed similar patterns between WT and *PANS-CmDFR-RNAi* plants, except for *CmUFGT*, which showed significant decreases in both transgenic chrysanthemum plants.

Taken together, our expression analysis indicated that *CmDFR* RNAi under the control of a flower-specific promoter was effective at suppressing *CmDFR* expression and affected the flavonoid biosynthetic pathway at the transcriptional level in chrysanthemum ray florets, but had little or no effect in leaves.

### 2.4. Total Flavonoid Contents are Increased in PANS-CmDFR-RNAi Ray Florets

**Figure 6:**
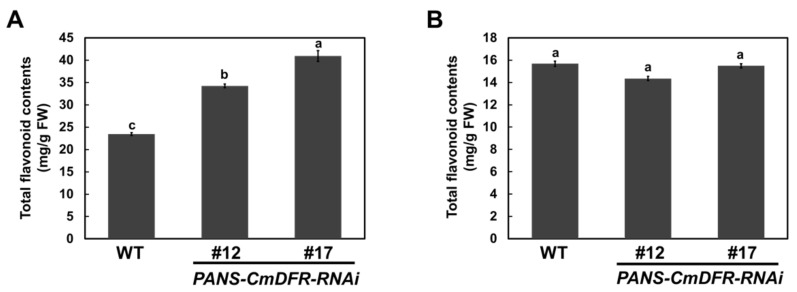
Total flavonoid contents in WT and *PANS-CmDFR-RNAi* transgenic chrysanthemum ray florets (**A**) and leaves (**B**). All results represent mean values ± SD from three independent biological replicates. Different letters above the bars indicate significantly different values (*p* < 0.01) calculated using one-way ANOVA followed by Duncan’s multiple range tests.

To assess total flavonoid contents under *CmDFR* suppression, we compared ray florets and leaves from WT and *PANS-CmDFR-RNAi* plants. Total flavonoid contents were higher in ray florets than in leaves of both WT and transgenic chrysanthemum lines (Figure 6). Interestingly, total flavonoid levels in ray florets were significantly higher in *PANS-CmDFR-RNAi* plants relative to WT plants. Specifically, *PANS-CmDFR-RNAi* plants contained 146~174% of the content of total flavonoids in WT plants. In leaves, total flavonoid contents of *PANS-CmDFR-RNAi* plants were similar to those of WT plants. These results were consistent with the expression patterns of flavonoid biosynthetic genes in ray florets and leaves. As shown in Figure 5, suppression of *CmDFR* under the control of a flower-specific promoter increased the expression of flavonoid biosynthetic genes in ray florets but not in leaves, resulting in elevated levels of total flavonoids in ray florets. Taken together, these results suggest that blockage of downstream metabolites through post-transcriptional silencing of the *CmDFR* gene can modulate transcript levels of flavonoid biosynthetic genes and the metabolic flux of flavonoid classes.

### 2.5. Antioxidant Activity is Enhanced in Ray Florets of PANS-CmDFR-RNAi Chrysanthemum

**Figure 7:**
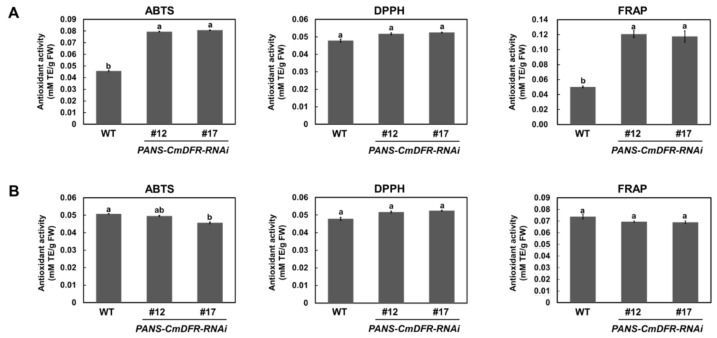
Total antioxidant activity in ray florets and leaves of WT and transgenic chrysanthemum plants. Total antioxidant capacity was measured using ABTS, DPPH, and FRAP assays in ray florets (**A**) and leaves (**B**). The results represent mean values ± SD from three independent biological replicates. Different letters above the bars indicate significantly different values (*p* < 0.01) calculated using one-way ANOVA followed by Duncan’s multiple range tests. The results are reported as Trolox equivalents (TE) per g of fresh weight (FW).

To evaluate the influence of flavonoids on antioxidant activity, we measured total radical-scavenging activities in extracts of ray florets and leaves from WT and *PANS-CmDFR-RNAi* plants using 2,2′-azinobis (3-ethylbenzothiazoline-6-sulfonic acid) diammonium salt (ABTS), 1,1-diphenyl-2-picrylhydrazyl (DPPH), and ferric ion-reducing antioxidant power (FRAP) assays (Figure 7). Antioxidant activities were higher in ray florets of *PANS-CmDFR-RNAi* plants than in those of WT plants, with levels ranging from 176% to 173% (ABTS), 108% to 109% (DPPH), and 239% to 233% (FRAP) those in WT plants, respectively. However, antioxidant activities in leaf extracts were similar in WT and *PANS-CmDFR-RNAi* plants. These results coincided well with the total flavonoid contents, suggesting that altering total flavonoid contents through silencing *CmDFR* greatly enhanced the total antioxidant capacity of ray florets.

## 3. Discussion

Chrysanthemum is an important ornamental and medicinal plant species. To date, molecular and genetic investigations have largely focused on flowering time and abiotic stress of the ornamental types [26,27,28]. However, little is known about the metabolic flux of flavonoid biosynthesis in chrysanthemum.

To investigate the role of DFR in flavonoid biosynthesis, we constructed an RNAi vector harboring the conserved region of previously reported chrysanthemum seven *CmDFR* genes. Transgenic *PANS-CmDFR-RNAi* chrysanthemum plants showed dramatically down-regulated expression of *CmDFR* genes in ray florets without any other morphological differences compared with WT plants (Figure 2). Interestingly, *PANS-CmDFR-RNAi* chrysanthemum ray florets showed elevated expression of the genes encoding FNS and FLS, which are important for metabolic channeling toward flavone and flavonol biosynthesis, respectively (Figure 5). Post-transcriptional gene silencing of *FNS* leads to excessive accumulation of anthocyanins, resulting in a black color in dahlia flowers [28]. An inverse relationship between flavone and anthocyanin contents is observed among red and black dahlia cultivars [10,29]. Additionally, ectopic expression of celery *FNS* leads to repression of *F3′H*, *DFR*, and *UFGT*, resulting in higher levels of flavones and lower levels of anthocyanins [18]. Similarly, ectopic expression of *DFR* enhances anthocyanin accumulation and reduces flavonol accumulation in *Arabidopsis* and camellia (*Camellia nitidissima*) [19,30]. By contrast, suppression of *DFR* leads to higher flavonol contents and lower anthocyanin levels in *Arabidopsis*, tobacco, and petunia (*Petunia hybrida*) [31,32,33]. Alongside these previous results, our current findings show that altering *DFR* expression modulates the expression of genes in the flavonoid biosynthetic pathway, thus altering the accumulation of different classes of flavonoids.

Flavonoid enzymes physically interact with each other to form a metabolon with cytochrome P450 [12,34]. For example, endoplasmic reticulum-bound cytochrome P450 enzymes, such as FNS and IFS, serve a crucial role in metabolon formation and contribute to the biosynthesis of different classes of flavonoids. The temporal interaction pattern of flavonoid enzymes is consistent with flavonoid patterns in plants including snapdragon, torenia, and soybean (*Glycine max*) [12,13,34]. In snapdragon, expression of *FNS* is a prerequisite for flavonoid metabolon conformation; FNS functions in a structural hub without catalytic ability to bind soluble enzymes such as CHS, CHI, and DFR, resulting in metabolic flux into anthocyanin biosynthesis in a flower stage-dependent manner. Similar to the situation in snapdragon, the protein–protein interaction between FNS and DFR has been reported in torenia and the suppression of *FNS* decreased the flavone and anthocyanin contents in petals [12]. In soybean, the interaction of CHS and chalcone reductase with IFS leads to the production of deoxyisoflavonoids in roots and seeds, indicating that the formation of metabolon complexes determines the micro-compartmentalization of flavonoid metabolites [34].

Previous studies have reported that the tobacco flower-specific promoter *PANS* can restrict gene expression to floral tissues [35,36]. In this study, dramatic suppression of the *CmDFR* gene was detected in ray florets but not in leaves, verifying that *PANS* works exclusively in floral tissues of heterologous chrysanthemum plants (Figure 5). Additionally, ray florets of *PANS-CmDFR-RNAi* chrysanthemum accumulated higher levels of total flavonoids and displayed higher antioxidant activity than those of WT plants, paralleling levels of *FLS* and *FNS* expression (Figure 5, Figure 6 and Figure 7). These results suggest that transcriptional regulation of flavonoid genes, associated with the expression level of *DFR* genes, is responsible for the redistribution of metabolic flux as a component of positive feedback and feedforward mechanisms.

## 4. Materials and Methods

### 4.1. Vector Construction

To down-regulate *CmDFR* gene expression in chrysanthemum, an RNAi-inducing vector was constructed using the conservative region from all annotated *CmDFR* sequences. For the *CmDFR-RNAi* construct, a fragment of the *CmDFR* coding region (567 bp, corresponding to nucleotides 513 to 1080) was amplified by PCR using primer set CmDFR-sense F/R. The PCR fragment was subcloned between the attL1 and attL2 regions of *EcoR*I-digested pUC57 mini using the InFusion Cloning System (Takara, Otsu, Japan). The resulting plasmid was then digested with *EcoR*I and combined in antisense orientation with a 338 bp fragment amplified by PCR using primer set CmDFR-antisense F/R.

The resulting pUC57 mini-*CmDFR-RNAi* construct was incorporated into the gateway destination vector pB2WG7 (VIB-Ghent University, Ghent, Belgium). To generate recombinant constructs driven by a flower-specific promoter, the *CaMV 35S* promoter region in the pB2WG7-*CmDFR-RNAi* construct was replaced by the previously reported flower-specific promoter *PANS* via PCR using primer set PANS-F/R [34,35]. The resulting vector, *PANS-CmDFR-RNAi* (Figure 1), was transferred into *Agrobacterium* strain LBA4404 using the freeze–thaw method for chrysanthemum transformation. All primers used in this study are listed in Supplementary Table S1.

### 4.2. Agrobacterium-Mediated Transformation of Chrysanthemum ‘BaekGang’

In this study, Chrysanthemum cultivar ‘BaekGang’ was obtained at the National Institute of Horticultural and Herbal Science (Wanju, Korea) and used for *Agrobacterium*-mediated transformation as described previously [22]. Briefly, fully expanded leaves from in vitro cultured plantlets were detached and cut into square segments of approximately 8–10 mm. Leaf discs were submerged in *Agrobacterium* harboring *PANS-CmDFR-RNAi* for 20 min under gently shaking and blotted dry using sterile filter paper. Inoculated explants were then co-cultivated in the dark for 3 days on shoot induction medium (SIM) supplemented with 100 μM acetosyringone. SIM consisted of MS salts and vitamins, 30 g·L^−1^ sucrose, 0.5 mg·L^−1^ 6-benzylaminopurine, 0.5 mg·L^−1^ naphthaleneacetic acid, and 7 g·L^−1^ agar. After co-cultivation, explants were placed on transgenic shoot induction medium (TSIM) consisting of SIM supplemented with 0.75 mg·L^−1^ PPT and 250 mg·L^−1^ cefotaxime. Explants were cultured for 5 weeks under dark conditions and subsequently for 4 weeks under light conditions (16-h light/8-h dark) at 25 ± 2 °C with a PPFD of 100 µmol m^−2^ s^−1^ using metal halide lamp. After 9 weeks on TSIM, regenerated shoots were detached from explants and cultured on transgenic shoot elongation medium (TSEM) consisting of TSIM without hormones. Surviving shoots were sub-cultured three to four times to fresh TSEM medium at 4-week intervals. Shoots longer than 0.5 cm were transferred to root induction medium. Plantlets with well-developed roots were planted in small plug trays for acclimation and finally transferred into pots filled with bedding soil. For further study, putative transgenic chrysanthemum lines were propagated as stem cuttings.

### 4.3. Genomic DNA Isolation and PCR

For confirming the presence of the transgene in putative three transgenic plants and stable transgene transfer via stem cuttings, genomic DNA was extracted from chrysanthemum leaves using a Plant Mini Kit (Qiagen, Valencia, CA, USA) according to the manufacturer’s instructions. PCR was performed using primer set PANS-F and CmDFR-sense-R for the presence of the transgene and primer set CmDFR-ORF-F/CmDFR-sense-R for the intactness of genomic DNA. PCR was performed with a C1000 touch cycler (Bio-Rad Laboratories, Hercules, CA, USA) with the conditions as follows: denaturation at 98 °C for 2 min, followed by 30 cycles of 98 °C for 10 s, 68 °C for 30 s, and extension at 72 °C for 5 min. PCR products were visualized using 1% (*w*/*v*) agarose gel electrophoresis followed by ethidium bromide staining.

### 4.4. Total RNA Extraction and qRT-PCR Analysis

Total RNA was extracted from ray florets of flowers and leaves from WT and transgenic chrysanthemum using Plant RNA Purification Reagent (Invitrogen, Carlsbad, CA, USA) and pretreatment with Fruit-mate for RNA Purification solution (Takara), as described previously [8,9]. Total RNA was purified using a FavorPrep Plant Total RNA Mini Kit (Favorgen, Changzhi, Taiwan), according to the manufacturer’s instructions. cDNA was synthesized from 2 µg of total RNA using amfiRivert cDNA Synthesis Platinum Master Mix (GenDEPOT, Barker, TX, USA).

qRT-PCR was performed using AccuPower 2x Greenstar qPCR Master Mix (Bioneer, Daejun, Korea) and a Bio-Rad CFX96 Detection System (Bio-Rad Laboratories), according to the manufacturers’ instructions. Gene expression was normalized using *elongation factor 1α* (*EF1α*) as a reference gene. Gene-specific primers used for qRT-PCR analysis are listed in Supplementary Table S1. Three independent biological replicates were performed per sample.

### 4.5. Measurement of Total Flavonid Content

Total flavonoid content was determined with ray florets and leaves from WT and transgenic chrysanthemum as described previously [32]. Briefly, powdered samples were incubated in 1 mL 50% (*v*/*v*) methanol for 6 h at room temperature with moderate shaking. Total flavonoid contents were determined by adding 0.5 mL of extract to 5 mL of diethylene glycol and 0.5 mL of 1 M NaOH. The mixture was shaken thoroughly and allowed to stand for 1 h at 37 °C. The absorbance of the solution was measured at 420 nm. Total flavonoid contents were determined using a standard curve obtained from various concentrations of naringin.

### 4.6. Antioxidant Activity Assays

The ABTS, DPPH, and FRAP assays were performed as described previously [32]. For the ABTS assay, 7 mM ABTS was mixed with 2.45 mM potassium persulfate and the mixture was stored for 12 h in the dark at room temperature. The solution was diluted until the absorbance at 734 nm reached 1.1 ± 0.02 as measured using a microplate reader. Leaf and ray floret extracts (10 μL) from WT and transgenic chrysanthemum plants were mixed with 300 μL of diluted ABTS solution in 96-well plates and incubated for 30 min in the dark. The absorbance was then measured at 734 nm using a microplate reader (μQuan BioTek Instruments, Winooski, VT, USA).

For the DPPH assay, leaf and ray floret extracts (10 μL) from WT and transgenic chrysanthemum plants were mixed with 300 μL of DPPH ethanol solution (0.2 mM) in 96-well plates and incubated for 30 min at room temperature in the dark. The absorbance was then measured at 515 nm using a microplate reader.

For the FRAP assay, FRAP reagent was freshly prepared by mixing acetate buffer (pH 3.6), 10 mM TPTZ (in 40 mM HCl), and 20 mM FeCl_3_6H_2_O (in distilled water) at a ratio of 10:1:1. Leaf and ray floret extracts (10 μL) from WT and transgenic chrysanthemum plants were mixed with 300 μL of FRAP solution in 96-well plates and incubated for 30 min at 37 °C. The absorbance was then measured at 593 nm using a microplate reader. All of the experiments were performed in triplicate, and all of the results are presented as mM trolox equivalent per g of fresh weight.

### 4.7. Statistical Analysis

All the experiments of gene expression, total flavonoid content, and antioxidant activity were performed independently with three replicates. All data were expressed as the mean ± standard deviation. Different letters above the bars indicate significantly different values (*p* < 0.01) calculated using one-way ANOVA followed by Duncan’s multiple range tests.

## 5. Conclusions

In this study, we identified the role of *CmDFR* suppression on flavonoid biosynthesis. Post-transcriptional gene silencing of *CmDFR* under flower-specific promoter PANS can modulate the transcript level of flavonoid biosynthetic genes in ray florets, not in leaves. The total flavonoid content was higher in ray florets of *CmDFR*-RNAi plants than those of WT plants, but that was similar in leaves of both *CmDFR*-RNAi and WT plants. In parallel with total flavonoid content, antioxidant activity was enhanced in the ray florets of *CmDFR*-RNAi plants, but similar in leaves. These results indicated that the suppression of *CmDFR* gene can modulate the transcript level of flavonoid biosynthetic genes, resulting in enhanced total flavonoid content and antioxidant activity. These results suggested that modulation of *CmDFR* gene can be a good candidate for enhancing the antioxidant property via modifying the metabolic flux of flavonoid biosynthesis as well as for precise genome editing for the development of the health benefit medicinal crop.

## Data Availability

The data presented in this study are available on request from the corresponding authors. The data are not publicly available because their elaboration are all reported in the manuscript.

## Supplementary Materials

The following supporting information can be downloaded at: https://www.mdpi.com/article/10.3390/plants11131681/s1, Table S1: List of primers used in this study, Figure S1: Multiple alignments of the cDNA sequences of *CmDFR* genes from previously deposited at NCBI.
