# Supplementary material for: Silencing of Dihydroflavonol 4-reductase in Chrysanthemum Ray Florets Enhances Flavonoid Biosynthesis and Antioxidant Capacity
Source: Plants (Basel). 2022 Jun 24;11(13):1681. doi: 10.3390/plants11131681 (PMC9269342; doi:10.3390/plants11131681)
Supplement: Supplementary file 1 [file plants-11-01681-s001.zip › Supplementary Table and Figure.pdf]

Table S1. Gene specific primers used in this study.

Usage	Primer name	Primer sequence (5'-3')
Gene cloning	CmDFR-sense-F	AAAGCAGGCTGAATTCGGAGAAAGCAGCATGGAAA
	CmDFR-sense-R	AAAGCTGGGTGGATCCGCCACCTTATGGATAATCTG
	CmDFR-antisense-F	ATAAGGTGGCGGATCTTAGTTGGAACTTGATACTC
	CmDFR-antisense-R	AAAGCTGGGTGGATCCGGAGAAAGCAGCATGGAAA
	PANS-F	ATGTATGATAATTCGAGCTCCGGTCAACTCAAGACAAC
	PANS-R	TTGTGATATCACTAGCTCTGTATTATTCTTGCTTTTT
Genotyping	PANS-F	ATGTATGATAATTCGAGCTCCGGTCAACTCAAGACAAC
	CmDFR-sense-R	AAAGCTGGGTGGATCCGCCACCTTATGGATAATCTG
	CmDFR-ORF-F	GCAGCATGGAAAGCAACAAAG
Gene expression	CmDFR-F	GCAGCATGGAAAGCAACAAAG
	CmDFR-R	GGGACTGATAAATGGACCAACAAC
	CmCHS-F	GGAGAAGATGAGAGCCACTAGACAC
	CmCHS-R	CAGGACCGAACCCGAATAAA
	CmCHI-F	GCAGGTGTGAGAGGTATG
	CmCHI-R	GCAACGGAATCGCTTTATC
	CmF3H-F	GGTGCAAGATTGGAGGGAGAT
	CmF3H-R	CAGGTTGAGGACACTTTGGGTAG
	CmFNS-F	GCCCAGTCCTCCATCGTTAC
	CmFNS-R	TAGCGGACCATAGCGAGTTGA
	CmFLS-F	TCCCATGGTTCACCACTTGA
	CmFLS-R	TAGTGGAGTCGTGTTTGGTA
	CmF3'H-F	TCAACGCCACTCTCCTTACCA
	CmF3'H-R	ACATCAGCACCAGGCTTTTCTC
	CmANS-F	CACCCCTCTTTCCGCCACGAACCTT
	CmANS-R	ATGTATTGTCTTATTATTCATGATG
	CmUFGT-F	TTCCCTTTTGCCTCACACCC
	CmUFGT-R	TTAAGAACCCTGCGAAACTCCT
	CmEF1α-F	TTTTGGTATCTGGTCCTGGAG
	CmEF1α-R	CCATTCAAGCGACAGACTCA

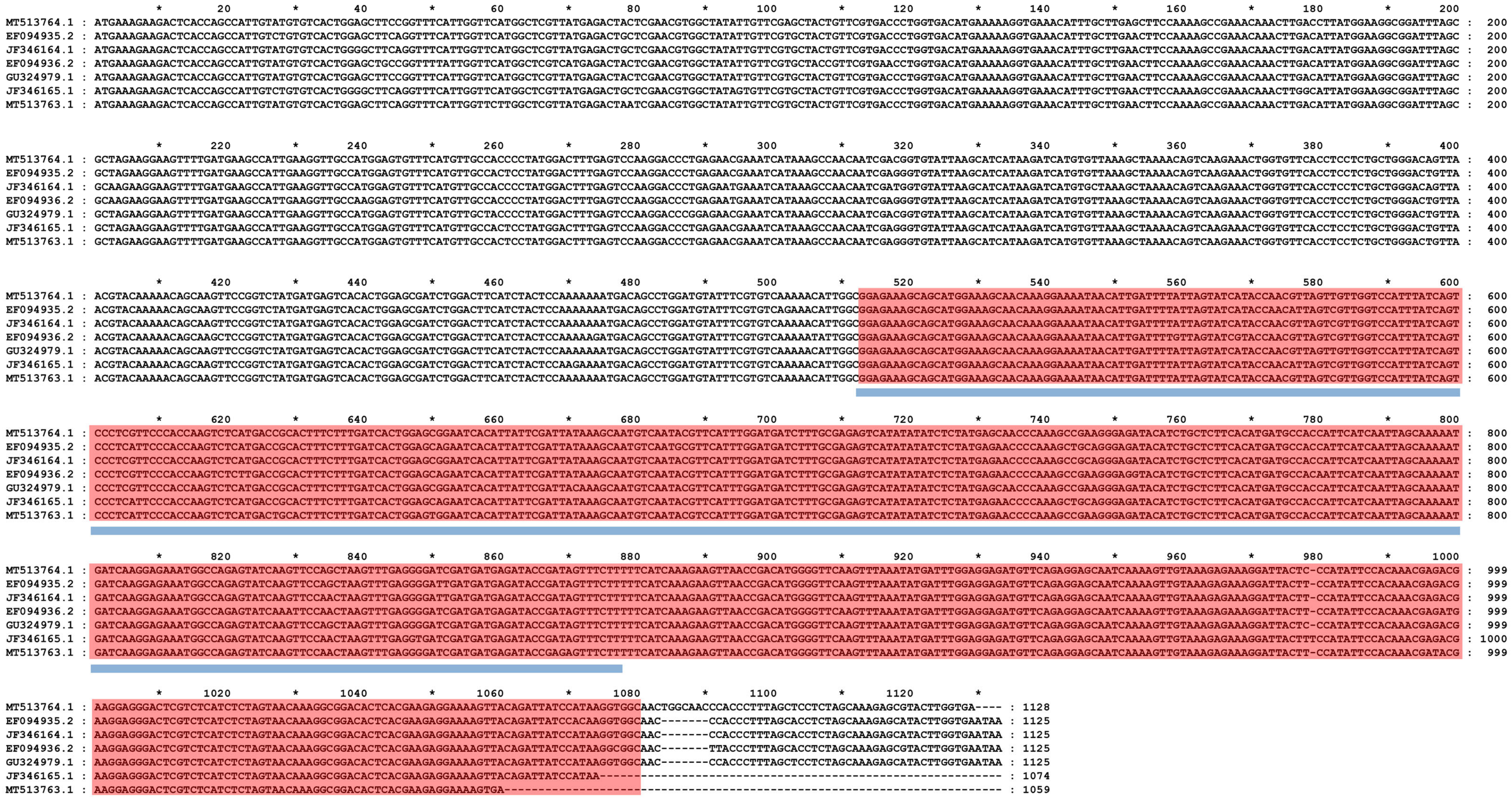

Figure S1. Multiple alignments of the cDNA sequences of *CmDFR* genes from previously deposited at NCBI. To gene silencing of DFR gene by RNA interference construct, the red box fragment was combined in the sense orientation with the antisense fragment which was indicated by the thick blue line.
